# Influenza antivirals currently in late‐phase clinical trial

**DOI:** 10.1111/irv.12446

**Published:** 2017-02-28

**Authors:** Paulina Koszalka, Danielle Tilmanis, Aeron C. Hurt

**Affiliations:** ^1^ WHO Collaborating Centre for Reference and Research on InfluenzaVIDRLPeter Doherty Institute for Infection and ImmunityMelbourneVictoriaAustralia; ^2^ University of MelbourneMelbourne School of Population and Global HealthParkvilleVictoriaAustralia

**Keywords:** antiviral, clinical trial, development, effectiveness, influenza, influenza virus

## Abstract

Influenza antiviral drugs are important for the control of influenza, most specifically for the treatment of influenza patients with severe disease following infection with a seasonal influenza virus, a newly emerging influenza strain, or in the event of a pandemic. Many influenza antivirals that are currently under investigation in late‐stage clinical trials differ in their mechanism of action compared to drugs currently licensed for the treatment of influenza. Nitazoxanide and DAS181 target components of the host cell and alter the ability of the virus to replicate efficiently, while small molecule drugs such as T705, JNJ63623872 and S‐033188 bind to the viral polymerase complex and restrict viral replication. Monoclonal antibodies that are currently in clinical trial for the treatment of influenza most commonly are targeted to the stem region of the haemagglutinin molecule. Early findings from animal models and in vitro studies suggest that many of the new antiviral drugs when tested in combination with oseltamivir have improved effectiveness over monotherapy. Clinical trials assessing both monotherapy and combination therapy are currently under investigation. It is hoped that as new antivirals are licensed, they will improve the standard of care and outcomes for influenza patients with severe disease.

## 1 Introduction

Although influenza vaccines remain the most effective means to prevent seasonal influenza, they typically provide suboptimal protection in high risk groups.^1^ In addition, vaccines may be completely ineffective in the event of an antigenic mismatch between viruses in the seasonal vaccine and those circulating in the community.^2^, ^3^ In comparison, influenza antiviral drugs typically remain effective against antigenic drift variants or even newly emerged pandemic viruses as they target highly stable or conserved parts of the virus. In many countries, antiviral medication is administered to hospitalised patients with severe influenza illness and is stockpiled as part of pandemic preparedness plans.^4^, ^5^, ^6^ Some countries, including Japan and the USA, also use substantial amounts of influenza antivirals for the treatment of uncomplicated influenza infection in otherwise healthy individuals.^7^, ^8^ Compounds from only two classes of influenza antivirals are licensed for use in many countries. These are the adamantanes and neuraminidase inhibitors (NAIs). The adamantane compounds, amantadine and rimantadine, target the viral M2 protein required for virus uncoating during replication.^9^ However, these compounds are no longer recommended for treatment of seasonal influenza A viruses due to widespread viral resistance.^10^ NAIs impair the release of virus from infected host cells, and although the frequency of NAI resistance in currently circulating strains is low (<1%),^11^ resistance to oseltamivir, the most widely used NAI, was widespread amongst former seasonal H1N1 viruses in 2008.^12^, ^13^ More recently, localised clusters of oseltamivir‐resistant H1N1pdm09 viruses have been detected.^14^, ^15^ Aside from concerns regarding resistance, the effectiveness of the NAIs is limited when delivered >48 hours after the onset of symptoms. Given these factors, there remains an important need for the development of influenza antiviral drugs that improve treatment effectiveness and can be delivered easily to patients with severe illness, remain effective when delivered late in the progression of disease and that have a low propensity for selecting for viral resistance.

This review summarises the new influenza antiviral drugs currently in phase II or III clinical development (as described on www.clinicaltrials.gov). This includes two compounds (nitazoxanide (NTZ) and DAS181 (Fludase)) that target features of the host cell that are critical for influenza replication, three compounds (T705 (favipiravir), JNJ63623872 (VX‐787) and S‐033188) that target components of the viral polymerase complex and a number of monoclonal antibodies that are being investigated as therapeutics in the treatment of influenza virus infection. A list of these antivirals including their mechanism of action and clinical trial status is provided in Table 1. Adjunctive therapies such as macrolides, non‐steroidal anti‐inflammatory drugs and mTOR inhibitors that have been investigated in combination with antiviral therapy are not discussed in this review.

**Table 1:** Summary of influenza antivirals currently in phase II or III clinical trials

Host/Viral targeted	Name	Type of antiviral	Specific target	Administration Route	Clinical trial phase	Manufacturer/Research Group
Host targeting	DAS181–F03/F04^a^	Sialidase	Neu5Ac α(2,3)‐ and Neu5Ac α(2,6)‐Gal linkages of sialic acid	Oral, inhalation	I, II	Ansun Biopharma, USA
	Nitazoxanide	Thiazolide	Haemagglutinin maturation	Oral, tablet	III	Romark, USA
Viral targeting	JNJ‐63623872	PB2 Inhibitor	Small molecule inhibitor of PB2	Oral, tablet	I, II	Janssen, Belgium
	T705	RNA‐dependent RNA polymerase	Purine pseudobase (incorporates in viral RNA)	Oral, tablet	II, III	Toyama, Japan
	S‐033188	Cap‐dependent endonuclease inhibitor	Small molecule inhibitor of cap‐dependent endonuclease	Oral, tablet	III	Shionogi, Japan
	CR6261	Monoclonal antibody	HA stem	Intravenous	I, II	Crucell/Janssen
	CR8020	Monoclonal antibody	HA stem	Intravenous	I, II	Crucell/Janssen
	MEDI8852	Monoclonal antibody	HA stem	Intravenous	I, II	MedImmune, USA
	MHAA4549A	Monoclonal antibody	HA stem	Intravenous	II	Genentech, USA
	VIS410	Monoclonal antibody	HA stem	Intravenous	II	Visterra, USA

a F03 and F04 refer to formulations of DAS181. DAS181‐F03 and DAS181‐F04 are 10 μm particles, however, F04 differs via the addition of MgSO_4_.

## 2 Host Cell Targeting Compounds

### 2.1 Nitazoxanide

NTZ is a compound of the thiazolide class of broad‐spectrum antiviral drugs that is orally administered and rapidly deacetylated in the blood to the active metabolic form tizoxanide (TIZ).^16^ NTZ was originally developed and licensed as an antiprotozoal/helminth drug and remains the only licensed treatment for *Cryptosporidium* infections.^17^ In addition, NTZ/TIZ is also effective against a range of bacterial^18^ and viral infections.^19^ NTZ/TIZ was first identified to have the potential for antiviral properties during clinical trials for Cryptosporidium treatment in patients with AIDS.^20^ Since that time, NTZ/TIZ has been shown to inhibit various viruses including gastrointestinal viruses like rotavirus, and influenza viruses, hepatitis B and C viruses.^19^ The mode of action of NTZ/TIZ against these viruses appears to differ. For rotavirus, TIZ interferes with the VP7 structural protein formation, while for hepatitis C, TIZ aids the activation of protein kinase R (PKR) resulting in downstream activation of the innate immune pathway.^19^ For influenza, TIZ exerts its antiviral activity by interfering with the assembly of the viral haemagglutinin (HA).^21^ TIZ inhibits influenza virus replication by impairing the trafficking of the HA from the endoplasmic reticulum (ER) to the Golgi, preventing the exit of mature virus particles from the host cell.^22^ TIZ also inhibits HA maturation by blocking HA terminal glycosylation which occurs prior to endoglycosidase H digestion of the HA terminal.^16^ Second generation thialozide compounds that are structurally related to NTZ have also been shown to have broad‐spectrum antiviral activity. Some of these derivatives demonstrate improved bio‐availability compared to NTZ and were shown to stimulate innate immune responses to reduce HIV replication in vitro.^23^ One of the thialozide compounds, the amino ester prodrug derivative RM5061, is now undergoing Phase I clinical trials.^24^

In vitro studies have identified that NTZ/TIZ has antiviral activity against a range of different influenza A (H1, H3, H5, H7) and B viruses.^22^, ^25^, ^26^ In addition, a combination of oseltamivir and NTZ/TIZ in vitro has a synergistic effect against both human and avian influenza A viruses, including an oseltamivir‐resistant virus.^27^ The potential benefit in using a virus‐targeting antiviral (oseltamivir) in combination with the host‐targeting antiviral (NTZ) is the improvement in the effectiveness of influenza treatment as well as the reduction in the likelihood of selecting for resistance. There is currently no published data available on the effectiveness of NTZ treatment of influenza in animal models. Results from a phase IIb/III human clinical trial demonstrated that 600 mg NTZ twice daily (started within 48 hours of symptom onset) reduced symptom duration by 36 hours. Furthermore, this dosage regime also reduced the infectious viral load by up to one log_10_ compared to a placebo control.^28^ Four phase II or III clinical trials involving NTZ are currently underway, including a four‐arm comparison of oseltamivir vs NTZ vs oseltamivir/NTZ combination vs placebo (NCT01610245). Phenotypic analysis of viruses from day 1 and day 7 in patients being treated with NTZ found no significant change in NTZ susceptibility.^28^ This supports in vitro data which has shown that serial passaging of either influenza or hepatitis C viruses in the presence of increasing concentrations of NTZ failed to select for resistance.^25^, ^29^

### 2.2 DAS181 (Fludase)

DAS181 (also known as Fludase) is a host‐targeted recombinant sialidase fusion protein. It is designed to remove sialic acid receptors from glycan structures on the respiratory epithelium, thereby restricting the ability of influenza viruses to bind and enter the host cell. DAS181 is comprised of two main components, a heparin binding sequence, which anchors to respiratory epithelium, and a sialidase catalytic domain derived from *Actinomyoces viscous*. The active domain of the sialidase (which cleaves both α(2,6)‐linked and α(2,3)‐linked sialic acid receptors recognised by both human and avian/equine influenza viruses) is derived from a bacterial species that is also part of the human flora; therefore, administration is unlikely to produce adverse immunological reactions.^30^ The heparin binding sequence is derived from human amphiregulin; this component binds negatively charged glycosaminoglycans and improves the binding potency to the respiratory epithelium. DAS181 is administered as an inhaled dry powder with microparticles of 5‐10 μm in size, enabling the drug to access the upper and central respiratory tract, but not the lower respiratory tract. An early concern regarding the removal of sialic acid from the respiratory epithelium was that it may increase the colonisation rate of *Streptococcus pneumonia*, but to the contrary, studies have indicated that it decreases the risk of secondary bacterial infections.^30^ In vitro studies have shown that DAS181 treatment removed approximately 90% of sialic acid receptors on human airway epithelial (HAE) cells within 15 minutes and up to 15% of the dose was retained up to 7 days post‐treatment.^31^ At least 3 days was required for sialic acid receptor levels to return to normal.^31^

As a broad‐spectrum treatment of the respiratory epithelium, DAS181 has been shown to be active against a wide range of influenza viruses (H1N1pdm09, H3N2, H7N9, H5N1 and influenza B viruses), as well as parainfluenza viruses and human metapneumoviruses.^32^, ^33^, ^34^, ^35^ For the treatment of highly pathogenic H5N1, DAS181 was found to be effective in vitro in human lung tissue cultures and in a mouse model where 1 mg/kg/day given prophylactically was found to protect against H5N1 infection, and 0.7 mg/kg/day reduced mortality by 94%.^36^ Similar data were observed for the treatment of H7N9 viruses, including an oseltamivir‐resistant variant, where prophylactic administration of DAS181 completely protected mice from H7N9 infection, compared to untreated mice which died 9 days post‐infection. Treatment with 1 mg/kg/day DAS181 reduced viral lung titres significantly in both WT H7N9 and oseltamivir‐resistant H7N9 infected mice, compared to placebo‐treated controls.^37^ Interestingly, a 48‐hour delay in treatment still resulted in greater than 75% survival in H7N9 infected mice. Serial passage of influenza viruses in the presence of DAS181 selected for several mutations in the HA (G137R, S136T, S186I) and NA (W438L, L38P) which resulted in viruses with increased receptor binding, coupled with significantly reduced NA on the cell surface.^38^ As a result, the replication fitness of the DAS181 selected viruses was compromised.^38^ Four different formulations of DAS181, which differ in particle size, have been investigated in phase I and phase II trials. A phase II trial conducted over three influenza seasons examined the effectiveness of DAS181 F02 as a single 10 mg dose or 10 mg daily over 3 days. In mice receiving the multiple dose regimen, there was a significant decrease in viral shedding and viral load up to 5 days from the commencement of treatment.^39^ The safety of 20 mg doses of DAS181 F03 and F04 delivered for up to 10 days was assessed in a phase I trial. The dose was well tolerated up to 7 days, but further dosing resulted in the onset of adverse respiratory events.^40^ DAS181 has also been tested in a small cohort of adults with well‐controlled asthma. While the drug was associated with some adverse events such as chest discomfort, the drug was deemed safe for adults with uncomplicated asthma.^41^

## 3 Viral Polymerase Inhibitors

### 3.1 T705 (favipiravir)

T705 (also known as favipiravir) is a substituted pyrazine derivative which inhibits the replication of a number of RNA viruses including influenza viruses. As a purine nucleoside analogue, T705 exerts its antiviral activity as a competitive substrate inhibitor of the RNA‐dependent RNA polymerase (RDRP) that is essential for the replication of RNA viruses.^42^ Serial passage of viruses in increasing concentrations of T705 caused an increase in guanosine to adenine nucleotide mutations in the virus compared to viruses passaged in the absence of drug.^43^ The increased mutation frequency, resulting in the generation of non‐viable virus, is a key antiviral mechanism of T705.^43^ This has also been described as the mechanism of action for the antiviral ribavirin.^44^ T705 has broad‐spectrum activity, also targeting and successfully inhibiting the RDRP of other viruses in vitro*,* and in many cases in vivo*,* such as West Nile virus, poliovirus and noroviruses.^45^ In addition, T705 has also been shown to have some effectiveness against Ebola virus in small animal models^46^, ^47^ and was recently investigated as a treatment of severe Ebola virus disease in a phase II human clinical trial in West Africa (NCT02662855).

This broad‐spectrum antiviral viral agent has been shown to strongly inhibit the replication of human and avian influenza A viruses as well as influenza B and C viruses both in vitro and in vivo.^48^ T705 administration in mice results in a dose‐dependent reduction in both mortality and lung viral titres.^49^ A dose of 200 mg/kg/day treatment for 5 days prevented mortality in mice and reduced influenza H1N1 (A/PR/8/34) virus titres in the lung by 80% to below detectable levels.^49^ A study investigating treatment of mice infected with highly pathogenic H5N1 compared the effectiveness of oseltamivir or T705 treatment delivered either four times a day or as a single treatment. Treatment four times a day with T705 resulted in a very high survival rate and greatly reduced lung titres at all doses (300 mg/kg/day, 100 mg/kg/day and 33 mg/kg/day).^50^ Mice treated with 300 mg/kg/day t705 had approximately half the virus load of those treated with oseltamivir (at a dose of 20 mg/kg/day). Oseltamivir prevented death in 10%‐20% of mice, compared with 90%‐100% of T705 treated mice. A separate study showed that a 300 mg/kg dose of T705 resulted in nearly 100% survival rate in mice infected with H5N1, even when treatment was started 72 hours post‐infection, compared to mice given a 50 mg/kg dosage of oseltamivir which had a 50% survival rate.^51^

T705 has also been found to work synergistically with NAIs, improving survival, body weight loss and viral lung titres in mice compared to monotherapy.^42^ A recent study of combined treatment of oseltamivir (20 mg/kg/day) and T705 (50 mg/kg/day) resulted in 100% survival in mice infected with H5N1 and extended the treatment window to 48, 72 and 96 hours post‐infection. This combination therapy was more effective compared to treatment with oseltamivir alone (40% survival at 72 and 96 hours post‐infection) or T705 alone (90% and 40% survival at 72 and 96 hours post‐infection), respectively.^52^ Overall, these studies indicate that a single dose of T705 per day can protect against mortality in mice, and that when delivered as monotherapy or in combination with oseltamivir, T705 greatly extended the treatment window to achieve survival of mice infected with H5N1.

T705 has received a limited licensure in Japan, where it has been approved for use only in patients infected with novel or re‐emerging influenza viruses (ie in the event of a pandemic), and only when that virus is resistant to other influenza antivirals. It will only be manufactured and distributed upon request by the Minister of Health, Labor and Welfare in Japan.^53^ These conditions have been put in place due to concerns of teratogenicity, which have been identified in animal experiments.^54^ Two phase III studies of T705 monotherapy in the Americas, Europe and other countries have been completed, but at this stage, results are not publically available concerning the safety or efficacy of the drug. The susceptibility of H1N1pdm09 and H3N2 viruses taken from Japanese patients one and 2 days post‐T705 treatment found that no significant changes in T705 susceptibility were identified. However, a L666F amino acid substitution in the PA gene was detected in H1N1pdm09 viruses and shown to the reduce T705 activity on the viral polymerase.^55^

### 3.2 JNJ63623872 (formerly known as VX‐787)

JNJ63623872 (formerly known as VX‐787) is a compound that inhibits the PB2 subunit of influenza A viruses by utilising host pre‐mRNA as a primer for viral RNA synthesis during replication, a method known as “cap snatching”.^56^ The drug binds to key residues in the PB2 cap binding domain preventing the docking of the natural ligand, 7‐methyl GTP, thereby preventing viral RNA synthesis. The drug exhibits strong antiviral activity in vitro against influenza A viruses including highly pathogenic avian influenza viruses and those that are resistant to the neuraminidase inhibitors,^46^ but is ineffective against influenza B viruses due to the inherent differences in the structure of the PB2 caps. The addition of JNJ63623872 to in vitro cultures of influenza virus at the initiation of infection prevented viral RNA production, while the addition of the compound at six hours post‐infection stopped further production of positive sense viral RNA as well as reducing the production of negative sense viral RNA.^57^ Furthermore, JNJ63623872 was found to inhibit the transcription of viral RNA in human macrophages infected with influenza without interfering with cellular responses to infection. Metabolomic analysis indicated that the presence of the drug did not affect activation of cellular innate immune responses.^58^ JNJ63623872 has been shown to act synergistically with oseltamivir, zanamivir and favipiravir in vitro.^57^ JNJ63623872 was effective in both prophylaxis and treatment models of mouse influenza, including when treatment was delayed. Administration of the drug up to 96 hours post‐infection resulted in 100% survival and was superior to oseltamivir treatment in which 100% survival was only observed when mice were dosed up to 24 hours post‐infection.^46^ A comparison of the effectiveness of JNJ63623872 against H1N1pdm09 and H3N2 infection in a mouse model indicated that the drug was less active against H3N2 virus infection, with greater body weight loss and only 30% survival at the 2 mg/kg/day Dose compared to complete survival in H1N1pdm09 infected mice. In addition, a greater reduction in viral titres was observed in H1N1pdm09 infected mice compared to those infected with H3N2 virus.^59^

Variant influenza viruses with reduced susceptibility to JNJ63623872 have been selected in vitro at different concentrations of the drug. Six different amino acid substitutions in the PB2 (Q306H, S324I, S324N, S324R, F404Y and N510T) were detected in different viruses and demonstrated >10‐fold reduction in sensitivity to JNJ6362387.^60^ However, these PB2 substitutions are rarely observed in naturally occurring human isolates.^60^ In patients undergoing JNJ63623872 treatment in a human challenge study, a M431I amino acid substitution in PB2 was selected in viruses in four of 72 patients. These viruses showed a >50‐fold reduction in JNJ63623872 susceptibility and had reduced viral fitness.

A placebo‐controlled phase IIa study showed JNJ63623872 to be well tolerated and at the highest dose (1200 mg once daily for 5 days) resulted in a 94% reduction in viral shedding compared to placebo. The JNJ63623872 treatment group had a quicker resolution of influenza‐like symptoms (1.9 days) compared to the placebo group (3.7 days).^61^ Two phase II studies are ongoing, and the first (NCT02342249) is assessing 300 mg JNJ63623872 vs 600 mg JNJ63623872 vs 600 mg JNJ63623872 + 75 mg oseltamivir treatment arms in uncomplicated influenza adult patients. The other phase II trial (NCT02532283) is assessing the combination of 600 mg JNJ63623872 + 75 mg oseltamivir vs 75 mg oseltamivir alone in hospitalised adult and elderly patients with influenza A. A phase I trial is also evaluating the safety and pharmacokinetic interaction of JNJ63623872 with AL‐974, a PA inhibitor that is in early‐stage development.

### 3.3 S‐033188

S‐033188 is a prodrug that is metabolised to an active form (known as S‐033447). S‐033447 is a small molecule inhibitor of the cap‐dependent endonuclease of influenza A and B viruses. S‐033447 is highly effective at inhibiting a number of different influenza A and B viruses in vitro, while in a mouse model of infection, a single dose of S‐033188 was found to reduce viral load by 2 logs and reduced mortality compared to mice being treated with oseltamivir.^62^ In a phase II study of S‐033188, 400 adult patients with uncomplicated influenza were randomised to three different doses (10 mg, 20 mg and 40 mg) and compared to placebo. All three doses resulted in significant reductions in time to alleviation of symptoms and reduced virus titres at 24 and 48 hours post‐treatment compared to placebo.^62^

A phase III clinical trial (NCT02949011) to investigate the effect of a single dose of S‐033188 compared with placebo or oseltamivir 75 mg twice daily for 5 days in patients with influenza at high risk of complications is planned to begin recruitment in December 2016.

## 4 Monoclonal Antibodies

Several monoclonal antibodies (mAbs) are being developed as a treatment for severe influenza infection. Currently, five different mAbs which target the highly conserved stem region of the HA molecule of the virus and one targeted to the matrix protein are being evaluated in clinical trials. However, those summarised here are not an exhaustive list of all influenza mAbs in development. As the mAbs target conserved epitopes, they have the capacity to neutralise viruses across a broad range of influenza A subtypes. CR6261 is a mAb that binds to the helical region in the membrane‐proximal stem of HA1/HA2, enabling the antibody to neutralise a broad spectrum of influenza A viruses.^63^, ^64^ This mAb has also been shown to have beneficial prophylactic and therapeutic effects in ferrets infected with H5N1.^65^

Another mAb targeted to membrane fusion is CR8020, which binds to HA stem epitopes, is active against group 2 HA subtypes of influenza A viruses and has been shown to neutralise H3N2 and H7N7 infections in vitro and in vivo.^66^ CR8020 has been investigated in two placebo‐controlled phase II trials in experimentally infected individuals (NCT01938352) as well as in hospitalised patients with influenza, where it was also compared with the mAb CR6261 (NCT01992276). However, it has been shown that CR8020 targets residues that are prone to antigenic drift and therefore if escape mutants arise, then effectiveness is likely to be reduced.^67^

MEDI8852 also targets the HA stalk, but has a greater potency and a broader spectrum of activity showing effective inhibition of all 16 influenza A HA subtypes.^68^ MEDI8852 was shown to be superior to oseltamivir in preventing the death of mice and ferrets infected with H5N1 when treatment was administered up to 3 days post‐infection.^68^ MEDI8852 was shown to be well tolerated in phase I clinical trials,^69^ and patients are currently being recruited for a phase IIa study to evaluate its safety in patients with uncomplicated influenza (NCT02603952).

MHAA4549A is another HA stalk targeted mAb that is also effective at binding all influenza A subtypes.^70^ Pre‐clinical studies have shown efficacy in mice and ferrets challenged with H5N1 when treatment was delivered up to 72 hours post‐infection.^71^ Two phase I trials showed that the antibody was well tolerated as a single intravenous dose up to 10800 mg and had a mean half‐life of 22.5‐23.7 days.^72^ It was also effective as a therapy at high doses (3600 mg) in volunteers experimentally infected with A/Wisconsin/67/2005 (H3N2) virus (NCT01980966).^73^ Upcoming placebo‐controlled phase II trials will evaluate the mAb as a monotherapy in otherwise healthy adults with acute uncomplicated seasonal influenza (NCT02623322) and as a combination therapy with oseltamivir, compared with oseltamivir alone, in hospitalised influenza patients (NCT02293863).

Another HA mAb, VIS‐410, was effective at protecting mice from A(H7N9) virus challenge and improved viral clearance and spread in the lungs when delivered prophylactically. A single dose of 10 or 50 mg/kg protected all mice from death following infection with an oseltamivir‐resistant strain of influenza.^74^ In a phase I trial, 2‐50 mg/kg doses of VIS‐410 were found to be safe and well tolerated by the cohort.^75^

TCN032 differs from those previously discussed as it targets an epitope at the N‐terminus of the matrix 2 protein which is a conserved epitope in influenza A viruses. Binding to this target has been shown to inhibit almost all influenza virus types and protected mice from lethal challenges with either H5N1 or H1N1 influenza viruses.^76^ In a phase IIa human challenge study (NCT01719874), a single dose of TCN032 was found to be well tolerated.^77^

## 5 Conclusion

There is currently an unmet need for better influenza antiviral drugs for the treatment of patients with severe disease following influenza infection and to mitigate the impact of a pandemic. The availability of influenza antivirals with different targets (either viral or cellular) and consequently different modes of action provides multiple therapeutic options, including combinations of two or more compounds, that are likely to improve treatment effectiveness. In vitro and animal model data have shown the synergy of many of the new compounds with oseltamivir, and therefore, it is hoped that combination therapy will improve effectiveness in human clinical trials. Many of the new compounds are showing substantially improved effectiveness compared to currently available drugs when treatment is initiated >48 hours after symptom onset. In addition, the propensity for the selection of viral resistance to these new compounds appears to be lower than that seen for the NAIs or adamantanes. Finally, some of the new antivirals have activity against a number of different respiratory viruses which could mean that these broad‐spectrum compounds may be prescribed for a respiratory disease without the need for a specific laboratory confirmed diagnosis.
